# Relación entre la muerte y el ingreso a cuidados intensivos de pacientes pediátricos con bacteriemia por *Staphylococcus aureus* adquirido en la comunidad, 2014-2017

**DOI:** 10.7705/biomedica.5275

**Published:** 2020-10-23

**Authors:** Freddy Israel Pantoja, Willinton Robert Ricaurte, Diana Elizabeth Rosero

**Affiliations:** 1 Departamento de Medicina, Universidad de Nariño, Pasto, Colombia; 2 Hospital Infantil Los Ángeles, Pasto, Colombia

**Keywords:** *Staphylococcus aureus*, bacteriemia, muerte, morbilidad, cuidado intensivo, pediatría, *Staphylococcus aureus*, bacteremia, death, morbidity, critical care, pediatrics

## Abstract

**Introducción:**

La bacteriemia por *Staphylococcus aureus* adquirida en la comunidad (SA-AC) es una condición frecuente en pediatría que, además, constituye un problema de salud pública por las altas tasas de morbimortalidad y de resistencia bacteriana.

**Objetivos:**

Analizar los factores relacionados con la muerte y el ingreso a cuidados intensivos de pacientes menores de 18 años con bacteriemia por SA-AC que ingresaron al Hospital Infantil Los Ángeles de Pasto, Colombia, entre el 2014 y el 2017.

**Materiales y métodos:**

Se hizo un estudio observacional descriptivo y transversal. Se analizaron 86 pacientes con bacteriemia por SA-AC que cumplían los criterios de inclusión en el estudio utilizando un modelo multivariado de regresión logística.

**Resultados:**

El 25,6% de los 86 pacientes falleció y el 40,7% ingresó a la unidad de cuidados intensivos. La resistencia a la meticilina fue de 52,3%. Los focos principales de infección fueron los tejidos blandos, el sistema osteoarticular y el respiratorio. El 32,6% de los pacientes provenía de la zona del Pacífico de Nariño. Las etnias predominantes fueron la mestiza y la indígena. Entre los indígenas hubo mayor mortalidad que entre mestizos y afrocolombianos. En el análisis multivariado de la variable de muerte, se registró significación de la endocarditis (*odds ratio*, OR ajustado=20; IC_95%_1,5-254; p=0,02); no se registró significación estadística en cuanto al ingreso en la unidad de cuidados intensivos.

**Conclusiones:**

La bacteriemia por SA-AC determinó altas tasas de mortalidad e ingreso a la unidad de cuidados intensivos. Las cepas resistentes representaron el 52,3%, y la resistencia a la meticilina desembocó en una mayor mortalidad, aunque la mortalidad con cepas sensibles también fue considerable. La endocarditis fue responsable de una mortalidad bastante elevada. Se debe ajustar el tratamiento empírico cuando se sospeche bacteriemia por SA-AC.

## Abstract (en)

**Introduction:**

The bacteremia caused by *Staphylococcus aureus* acquired in the community (SA-AC) is a frequent pathology in pediatrics and it is considered a public health problem generating high rates of morbidity, mortality, and bacterial resistance.

**Objectives:**

To analyze the factors related to death and admission to intensive care units of patients under 18 years of age with AC-SA bacteremia admitted to the *Hospital Infantil Los Ángeles*, Pasto, Colombia, from 2014 to 2017.

**Material and methods:**

We conducted a descriptive, transversal, cross-sectional observational study. We analyzed 86 patients with bacteremia due to AC-SA that met the inclusion criteria for the study using a multivariate logistic regression model.

**Results:**

Of the 86 cases, 25.6% died and 40.7% entered the intensive care unit. The resistance to methicillin was 52.3%. The main foci of infection were the soft tissues and the osteoarticular and respiratory systems; 32.6% of patients came from the Pacific area of Nariño. The predominant ethnic groups were the mestizo and the indigenous. Indigenous patients had higher mortality compared to the mestizo and Afro-Colombian ethnic groups. The multivariate analysis showed significance in terms of death for endocarditis (adjusted OR=20; CI: 1.5-254; p=0.02) while no statistical significance was registered for the admission to the intensive care unit.

**Conclusions:**

The AC-SA led to high mortality and admission to the intensive care unit; 52.3% of strains were resistant and resistance to methicillin showed higher mortality, although the mortality with sensitive strains was considerable. Endocarditis showed fairly high mortality. The empirical therapy should be adjusted when bacteremia due to AC-SA is suspected.

*Staphylococcus aureus* es una bacteria Gram positiva conocida mundialmente por su alto grado de virulencia, lo cual determina su persistencia, recurrencia y tendencia a causar focos secundarios de infección (1,2). Se han descrito incidencias de bacteriemia de 4,6 a 8,4 casos por 100.000 niños, y del 9 al 12% de los pacientes con infecciones locales la desarrollan. Es especialmente agresiva en la población pediátrica y conlleva hospitalizaciones, tratamientos prolongados e ingreso a las unidades de cuidados intensivos hasta en el 33% de los casos, y la muerte, entre el 2,5 y el 8%, sobre todo con cepas de *S. aureus* resistentes a la meticilina (SARM), descritas en estudios de América y Europa en 3 a 30% de los pacientes (1-8).

Los factores relacionados con el ingreso a las unidades de cuidados intensivos y la muerte de pacientes con bacteriemia por SA-AC, se han estudiado poco en la población pediátrica, aunque se han descrito algunas características clínicas que pueden influir, como el foco de infección (neumonía), la desnutrición, la edad menor de un año, el sexo masculino, la prematuridad, la procedencia del área del Pacífico, la inmunodeficiencia, el eccema, un inadecuado tratamiento empírico y la presencia de SARM (3,5,9,10).

Son amplias las discrepancias en torno a la sensibilidad de *S. aureus* a la meticilina, la cual varía de un área geográfica a otra. Dado que la prevalencia de SARM adquirido en la comunidad viene en aumento desde hace 20 años, es necesario describir y documentar la epidemiología local de las cepas de la bacteria para contar con información útil en el momento del tratamiento empírico y disminuir complicaciones, como el ingreso a las unidades de cuidados intensivos y la muerte, debido a una selección inadecuada del antimicrobiano. Lastimosamente, no hay datos de este tipo en la zona del Pacífico colombiano y, menos, en el área de la pediatría (5).

En ese contexto, el objetivo de este estudio fue analizar los factores relacionados con la muerte y el ingreso a la unidad de cuidados intensivos de pacientes menores de 18 años con bacteriemia por SA-AC ingresados al Hospital Infantil Los Ángeles de Pasto entre 2014 y 2017.

## Materiales y métodos

**Figura 1:**
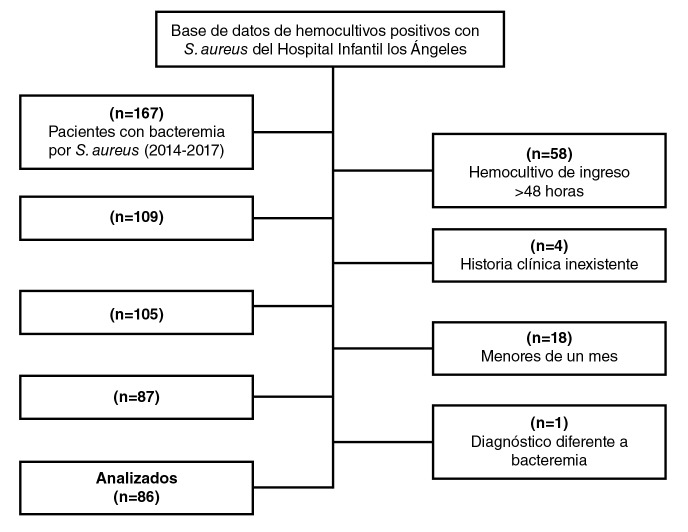
Flujograma de selección de los pacientes incluidos

Se hizo un estudio descriptivo y transversal mediante la revisión de las historias clínicas sistematizadas de todos (N=86) los pacientes mayores de un mes y menores de 18 años con dos hemocultivos positivos para *S. aureus* tomados por el personal del hospital bajo un protocolo estandarizado y procesados en la misma institución. No se hizo muestreo, ya que se incluyó toda la población atendida durante el período de estudio. Se excluyeron los pacientes diagnosticados con especies diferentes a *S. aureus*, aquellos cuya historia clínica estuviera incompleta, los que tuvieron hemocultivos positivos para *S. aureus* a partir de muestras tomadas después de 48 horas del ingreso al hospital y los remitidos de otra institución con una estancia hospitalaria superior a 48 horas (figura 1).

Se recopiló información sobre el ingreso a la unidad de cuidados intensivos, el estado vital y otras variables clínicas (sensibilidad de *Staphylococcus*, tratamiento, foco de infección, estado nutricional, estancia hospitalaria) y demográficas (sexo, edad, estrato socioeconómico, procedencia, zona de residencia, régimen de afiliación).

Se diseñó un instrumento tipo encuesta para recolectar la información, con preguntas cerradas sobre los datos de las historias clínicas sistematizadas. Se controlaron los sesgos de selección estableciendo criterios de inclusión y exclusión. Los sesgos de información se controlaron haciendo una búsqueda exhaustiva de las historias clínicas. Las definiciones de las variables se estandarizaron; se organizó el proceso de revisión de las historias clínicas, con el fin de no pasar por alto detalles o información valiosa, y se establecieron codificaciones para recopilar los datos de manera que mejorara su posterior procesamiento. Se hizo una prueba piloto con 20 pacientes para evaluar la capacidad de recolección de la información y la disponibilidad de los datos en las fuentes de información, y para validar el instrumento. No se excluyeron variables ni se imputaron datos.

Las variables cualitativas se resumieron en forma de frecuencias absolutas y relativas, y las cuantitativas, con medidas de tendencia central y dispersión. Se utilizó un modelo multivariado de regresión logística para determinar el riesgo de morir e ingresar a la unidad de cuidados intensivos (*odds ratio*, OR) de los pacientes con bacteriemia por *S. aureus* sensible a la meticilina, comparado con el riesgo de aquellos con cepas resistentes ajustado según las condiciones clínicas y demográficas. Todos los análisis se hicieron con un 95% de confianza en el programa SPSS™, versión 21.

## Consideraciones éticas

El estudio fue avalado por el Comité de Ética del Hospital Infantil Los Ángeles y el Comité de Ética de la Universidad de Nariño. Se clasificó como investigación ‘sin riesgo’ según el Artículo 11 de la Resolución 8430 de 1993 expedida por el Ministerio de Salud. El manejo de la información se ajustó a las normas establecidas por el Hospital para garantizar su uso con fines científicos y la confidencialidad de los datos personales de los pacientes.

## Resultados

### Variables demográficas y clínicas

Se revisaron 167 historias clínicas de pacientes mayores de un mes y menores de 18 años. Se detectaron 86 casos de bacteriemia por SA-AC y se reportó 25,6% (n=22) de mortalidad, 40,7% (n=35) de ingreso a la unidad de cuidados intensivos, 52,3% (n=45) de resistencia a la meticilina y 7% (n=6) de endocarditis. El sexo masculino representó el 67,4% (n=58) y la prevalencia fue mayor, 33,7% (n=29), entre escolares y lactantes. La edad promedio fue de 78 meses. Los focos clínicos de infección fueron los tejidos blandos, con 33,7% (n=29), el sistema osteoarticular, con 32,6% (n=28), el respiratorio, con 22,1% (n=19), el aparato digestivo, con 8,1% (n=7) y otros, con 3,5% (n=3).

Las zonas de procedencia de los pacientes fueron la región del Pacífico de Nariño, con 32,6% (n=28), y Putumayo, con 25,6% (n=22), seguido de otros, con 20,9% (n=18), de Pasto, con 12,8% (n=11), y de Ipiales, con 8,1% (n=7). La mayor prevalencia se registró en la zona rural, con 51,2% (n=44). El 13% (n=74) recibió antibiótico antes de la hospitalización; la etnia predominante fue la mestiza, con 59,3% (52), seguida de la población indígena, con 31,5% (n=27), y la afrocolombiana, con 9,3% (8). El 22,1% (n=19) de los pacientes presentaba desnutrición. El régimen de afiliación que predominó fue el subsidiado, con 94,2% (n=81).

### Relación de la muerte con las variables demográficas y clínicas

La mortalidad fue mayor entre los pacientes de etnia indígena, con 50% (OR crudo: 2,8; IC_95%_ 1-7,9; p=0,04). La resistencia a la meticilina determinó una mayor mortalidad, con 68,2%, frente a la cepa sensible, con 31,8% (OR crudo: 2,4; IC_95%_ 0,87-6,7; p=0,08); el 83,3% de los pacientes que desarrollaron endocarditis (7%; n=6), murió (OR crudo: 18; IC_95%_ 2-169; p=0,01), y en cuatro de ellos se aisló SARM. No se observó significación estadística en cuanto a sexo, procedencia, residencia, desnutrición, antibiótico previo o foco primario de infección (cuadro 1).

**Cuadro 1:** Relación de la muerte con las variables demográficas y clínicas de los pacientes con bacteriemia por *Staphylococcus aureus* adquirido en la comunidad, Pasto, Colombia, 2014-2017

		**Muerte**						***Odds ratio***	**p**
**Variable**		**Sí (n=22)**		**No (n=64)**		**Total (N=86)**		**(CI_95%_)**	
		**n**	**%**	**n**	**%**	**n**	**%**		
Variables demográficas									
Sexo	Femenino	7	32	21	33	28	33		0,93
	Masculino	15	68	43	67	58	67	1 (0,37-2,95)	
									
Edad	Adolescente	4	18,2	13	20,3	17	19,8		
	Escolar	8	36,4	21	32,8	29	33,7	1,2 (0,31-4,94)	0,76
	Preescolar	1	4,5	10	15,6	11	12,8	0,32 (0,03-3,37)	0,35
	Lactante	9	40,9	20	31,3	29	33,7	1,46 (,37-5,75)	0,59
									
Procedencia categorizada	Otros	4	18,2	14	21,9	18	20,9		
	Ipiales	2	9,1	5	7,8	7	8,1	1,39 (0,19-10,1)	0,74
	Pasto	3	13,6%	8	12,5	11	12,8	1,31 (0,23-7,4)	0,76
	Putumayo	6	27,3	16	25,0	22	25,6	1,31 (0,3-5,62)	0,71
	Pacífico	7	31,8	21	32,8	28	32,6	1,16 (0,28-4,74)	0,83
									
Residencia	Urbana	12	54,5	30	46,9	42	48,8		0,53
	Rural	10	45,5	34	53,1%	44	51,2	0,73 (0,27-1,94)	
									
Etnia	Mestizo	10	45,5	41	64,1	51	59,3		
	Indígena	11	50,0	16	25,0	27	31,4	2,8 (1-7,9)	0,04
	Afrocolombiano	1	4,5	7	10,9	8	9,3	0,58 (0,06-5,32)	0,63

Afiliación	Contributivo	3	13,6	2	3,1	5	5,8		
	Subsidiado	19	86,4	62	96,9	81	94,2	0,2 (0,032-1,31)	0,094
Variables clínicas									
Desnutrición	No	19	86,4	48	75,0	67	77,9		
	Sí	3	13,6	16	25,0	19	22,1	0,47 (0,12-1,81)	0,27
									
Antibiótico previo	No	19	86,4	55	85,9	74	86,0		0,96
	Sí	3	13,6	9	14,1	12	14,0	0,96 (0,23-3,94)	
									
Foco primario	Otros	1	4,5	2	3,1	3	3,5		
	Gastrointestinal	0	0,0	7	10,9	7	8,1	1,1 (0,08-13,8)	0,93
	Respiratorio	4	18,2	15	23,4	19	22,1	0,00	1,00
	Osteoarticular	8	36,4	20	31,3	28	32,6	0,59 (0,15-2,29)	0,45
	Tejidos blandos	9	40,9	20	31,3	29	33,7	0,88 (0,28-2,76)	0,84
									
Sensible a la meticilina	Sí	7	31,8	34	53,1	41	47,7		0,08
	No	15	68,2	30	46,9	45	52,3	2,4 (0,87-6,7)	
									
Endocarditis	No	17	77	63	98	80	93		
	Sí	5	23	1	2	6	7	18 (2-169)	0,01
									
Edad*		80	0,63	71	63	78	63	0,99 (0,99-1)	0,55
Edad*: media y desviación estándar									

### Relación del ingreso en la unidad de cuidados intensivos con las variables demográficas y clínicas

El 40% de los pacientes requirió ingreso a la unidad de cuidados intensivos. Se evidenció una relación entre la presencia de endocarditis y el ingreso a la unidad (OR: 80,9-74). El sexo masculino y la presencia de SARM prevalecieron, aunque no hubo una clara significación estadística (cuadro 2).

**Cuadro 2:** Relación del ingreso a la unidad de cuidados intensivos con las variables demográficas y clínicas de los pacientes con bacteriemia por *Staphylococcus aureus* adquirido en la comunidad, Pasto, Colombia, 2014-2017

		**Ingreso a cuidado intensivo**						***Odds ratio***	**p**
**Variable**		**Sí (n=35)**		**No (n=51)**		**Total (N=86)**		**(CI_95%_)**	
		**n**	**%**	**n**	**%**	**n**	**%**		
Variables demográficas									
Sexo	Femenino	14	40	14	28	28	33		0,22
	Masculino	21	60	37	73	58	67	0,56 (0,22 - 1,41)	
									
Edad	Adolescente	7	20	10	20	17	20		
	Escolar	13	37	16	31	29	34	0,1 (0,29 – 3,34)	0,98
	Preescolar	3	9	8	16	11	13	1,1 (0,4 – 3,25)	0,79
	Lactante	12	34	17	33	29	34	0,53 (0,11 – 2,42)	0,41
									
Procedencia categorizada	Otros	7	20	11	22%	18	21		
	Ipiales	4	11	3	6	7	8	2 (0,35 – 12,3)	0,4
	Pasto	4	11	7	14	11	13	0,89 (0,19 – 4,23)	0,89
	Putumayo	7	20	15	29	22	26	0,73 (0,19 – 2,70)	0,64
	Pacífico	13	37	15	29	28	33	1,36 (0,4 – 4,54)	0,61
									
Residencia	Urbana	19	54	23	45	42	49		0,4
	Rural	16	46	28	55	44	51	0,69 (0,29-1,64)	
									
Etnia	Mestizo	18	51	33	65	51	59		
	Indígena	13	37	14	28	27	31	1,7 (0,65 – 4,39)	0,27
	Afrocolombiano	4	11	4	8	8	9	1,8 (0,4 – 8,21)	0,42

Afiliación	Contributivo	3	9	2	4	5	6		
	Subsidiado	32	91	49	96	81	94	0,43 (0,06-2,75)	0,37
Variables clínicas									
Desnutrición	No	28	80	39	77	67	78		
	Sí	7	20	12	24	19	22	0,81 (0,28-2,32)	0,69
									
Antibiótico previo	No	30	86	44	86	74	86		0,94
	Sí	5	14	7	14	12	14	1 (0,3-3,61)	
									
Foco primario	Otros	2	6	1	2	3	4		
	Gastrointestinal	2	6	5	10	7	8	0,2 (0,01 – 3,6)	0,27
	Respiratorio	8	23	11	22	19	22	0,36 (0,02 – 4,7)	0,44
	Osteoarticular	9	26	19	37	28	33	0,23 (0,19 – 2,96)	0,26
	Tejidos blandos	14	40	15	29	29	34	0,46 (0,03 – 5,73)	0,55
									
Sensible a la meticilina	Sí	13	37	28	55	41	48		0,1
	No	22	63	23	45	45	52	2 (0,85-4,96)	
									
Endocarditis	No	30	86	50	98	80	93		
	Sí	5	14	1	2	6	7	8 (0,92-74)	0,058
									
Edad*		77	62	79	64	78	63	1 (0,99-1)	0,9
							

Edad*: media y desviación estándar

### Análisis multivariado

Se hizo un análisis multivariado para las variables con mayor significación en cuanto a la muerte y el ingreso a la unidad de cuidados intensivos, utilizando el test de Hosmer-Lemeshow, y se obtuvo un valor de p menor de 0,25. Se registró significación en cuanto al régimen de afiliación a los servicios de salud (OR ajustado: 0,122; IC_95%_ 0,016-0,906; p=0,04) y la endocarditis (OR ajustado: 20,136; IC_95%_ 1,596-254,094; p=0,02). Asimismo, la endocarditis pudo estar asociada con un factor relativo al ingreso a la unidad de cuidados intensivos (cuadro 3 y cuadro 4).

**Cuadro 3:** Análisis multivariado de muerte, Pasto, 2014-2017

**Variables**		**OR crudo**		**p**	**OR ajustado**		**p**
**CI_95%_**	**CI_95%_**
Etnia	Mestizo						
	Indígena	2,8	(1-7,9)	0,04	2,787	(0,83-9,3)	0,09
	Afrocolombiano	0,58	(0,06-5,3)	0,63	0,422	(0,03-6)	0,52
Afiliación	Contributivo	0,2	(0,032-1,3)	0,094	0,122	(0,01-0,9)	0,04
	Subsidiado						
Sensible a la meticilina	Sí	2,4	(0,8-6,7)	0,08	1,476	(0,4-4,6)	0,5
	No						
Endocarditis	No	18	(2-169)	0.01	20,136	(1,5-254)	0,02
	Sí						

**Cuadro 4:** Análisis multivariado de ingreso a la unidad de cuidados intensivos, Pasto, 2014-2017

**Variables**		**OR crudo**			**OR ajustado**		**p**
**CI_95%_)**	**p**	**CI_95%_**
Sexo	Masculino	0,56	(0,2-1,4)	0,22	0.53	(0,2-1,3)	0,2
	Femenino						
Sensible a la meticilina	Sí	2	(0,8-4,9)	0,1	1,85	(0,7-4,6)	0,18
	No						
Endocarditis	No	8	(0,9-74)	0,05	7	(0,7-64)	0,08
	Sí						

## Discusión

*Staphylococcus aureus* es uno de los principales agentes etiológicos de la bacteriemia, y causa una mortalidad de entre el 0,7 y el 8% e ingreso a la unidad de cuidados intensivos en 36% de los casos, sobre todo con cepas resistentes a meticilina adquiridas en la comunidad (4,6,11,12).

En el estudio, el 40% de los pacientes requirió hospitalización en la unidad de cuidados intensivos y el 25% falleció, cifras muy altas en comparación con otros estudios. Además, se evidenció SARM en 52% de los casos, lo que resulta preocupante dado el origen comunitario, siendo superior a los reportes por los grupos GREBO y GERMEN (30 y 22%) en Colombia y, en general, en América y Europa (2,9-23%), en cuyos países se ha evidenciado, además, una disminución de la frecuencia de estas cepas en varios estudios de seguimiento; no obstante, llama la atención que en ellos no se registró la diferencia estadística esperada entre las cepas resistentes y las sensibles en cuanto a la mortalidad y el ingreso a las unidades de cuidados intensivos pediátricos, tal como se ha hecho en algunos reportes, lo que sugiere una acentuada agresividad de *Staphylococcus* sensible a la meticilina adquirido en la comunidad (6,8,13).

Los focos de infección más frecuentes fueron los tejidos blandos y osteoarticulares, lo que coincide con lo reportado más frecuentemente en la literatura médica especializada (14,15).

La bacteriemia se presentó con mayor prevalencia en las regiones cálidas (Pacífico y Putumayo), lo que coincide con estudios en Paraguay y Nueva Zelanda (10,16). Los pacientes de etnia indígena presentaron mayor mortalidad y resistencia a la meticilina (OR crudo: 2,8; IC_95%_ 1-7,9; p=0,04), comparados con los de etnia mestiza o afrocolombiana, lo que sugeriría una mayor prevalencia de infecciones en ciertos grupos étnicos debido a que viven en comunidades más aisladas. En Nueva Zelanda, los estudios han demostrado que los niños de comunidades indígenas (maoríes) presentaron el doble de probabilidad de adquirir bacteriemia por *Staphylococcus* frente a los caucásicos de esa región (10). Aunque la endocarditis en pediatría no es una enfermedad frecuente, los informes mundiales indican una elevada mortalidad por esta causa, sobre todo cuando se aíslan cepas de *S. aureus*. En uno de los estudios, la mortalidad fue muy elevada y hubo una diferencia estadísticamente significativa con respecto a los pacientes sin endocarditis, lo que ya se ha evidenciado en estudios similares (6,17,18).

En nuestro hospital, el tratamiento empírico en pacientes con sospecha de bacteriemia por SA-AC debería dirigirse contra las cepas sensibles y las resistentes a la meticilina debido a su gran prevalencia y su asociación con la muerte, especialmente en indígenas y afrocolombianos procedentes de zonas como el litoral Pacífico y Putumayo. También, deben revisarse los tratamientos empíricos y los administrados en los casos de endocarditis por SARM causantes de gran mortalidad. Deben hacerse estudios analíticos capaces de generar asociaciones con la mortalidad.

Las limitaciones del estudio incluyeron el ser retrospectivo y basarse en el registro de historias clínicas de un solo hospital, algunas con datos incompletos. Asimismo, los datos no incluyeron la duración de la bacteriemia ni la mortalidad a largo plazo, pero es el primero que incluyó una considerable cantidad de pacientes y recabó información que previamente no se había considerado, lo que permite conocer la epidemiología local.
